# Supplementary material for: Kondo quasiparticle dynamics observed by resonant inelastic x-ray scattering
Source: Nat Commun. 2022 Oct 17;13:6129. doi: 10.1038/s41467-022-33468-6 (PMC9576770; doi:10.1038/s41467-022-33468-6)
Supplement: Supplementary file 1 — Supplementary Information [file 41467_2022_33468_MOESM1_ESM.pdf]

# Supplementary Information

## Kondo quasiparticle dynamics observed by resonant inelastic x-ray scattering

M. C. Rahn, K. Kummer, A. Hariki, K.-H. Ahn, J. Kuneš, A. Amorese, J. D. Denlinger, and D.-H. Lu, M. Hashimoto, E. Rienks, M. Valvidares, F. Haslbeck, D. D. Byler, K. J. McClellan, E. D. Bauer, J.-X. Zhu, C. H. Booth, A. D. Christianson, J. M. Lawrence, F. Ronning, and M. Janoschek

### Supplementary Notes

1. NCA/AIM calculations of $\chi''$
2. Comparison of RIXS and DFT+DMFT calculations of $\chi''$
3. Comparison of RIXS and inelastic neutron scattering
4. DFT+DMFT AIM-RIXS calculation of the Kramers-Heisenberg term
5. RIXS incident energy dependence and Pd $4d$ bands
6. RIXS incident energy and polarization dependence
7. Ce $M$-edge x-ray absorption
8. Overview of RIXS spectra and fits

## Supplementary Note 1: NCA/AIM calculations of $\chi''$

Calculations of the dynamic magnetic susceptibility $\chi''$ in the Anderson impurity model were performed using the large degeneracy or non-crossing approximation [1], as described in our earlier work [2]. This "toy model" of the hybridization phenomenon assumes a Gaussian conduction band of width $W$ centered at the Fermi surface $E_{\mathrm{F}}$, interacting with $4f^1$ states at the binding energy $E_f$, with a hybridization constant $V$, and spin-orbit–coupling $\Delta_{\mathrm{SO}}$. The calculations yield both static and dynamic quantities, such as the $f$-occupation number $n_f$, magnetic susceptibility $\chi$, and powder-averaged $\chi''$, and has proven universal in its applicability to $CePd_3$ [3] and other intermediate valence materials [4].

The input values for the background bandwidth $W$, the hybridization constant $V$, and the $f$-level energy $E_f$ are not obtained from first-principles calculations, nor do they necessarily reflect values taken from experiment. Rather, the calculation remains valid for the given experimental measurements as long as the universality of the Kondo physics holds, where the given quantities are universal functions of $T/T_{\mathrm{K}}$ or $E/(k_{\mathrm{B}}T_{\mathrm{K}})$. Since $T_{\mathrm{K}}$ depends exponentially on the ratio $(WE_f/V^2)$, the given parameters will reproduce the data as long as this ratio is approximately correct. To illustrate these characteristics, in Supplementary Figure 1, we show a comparison of calculations for two sets of parameters.

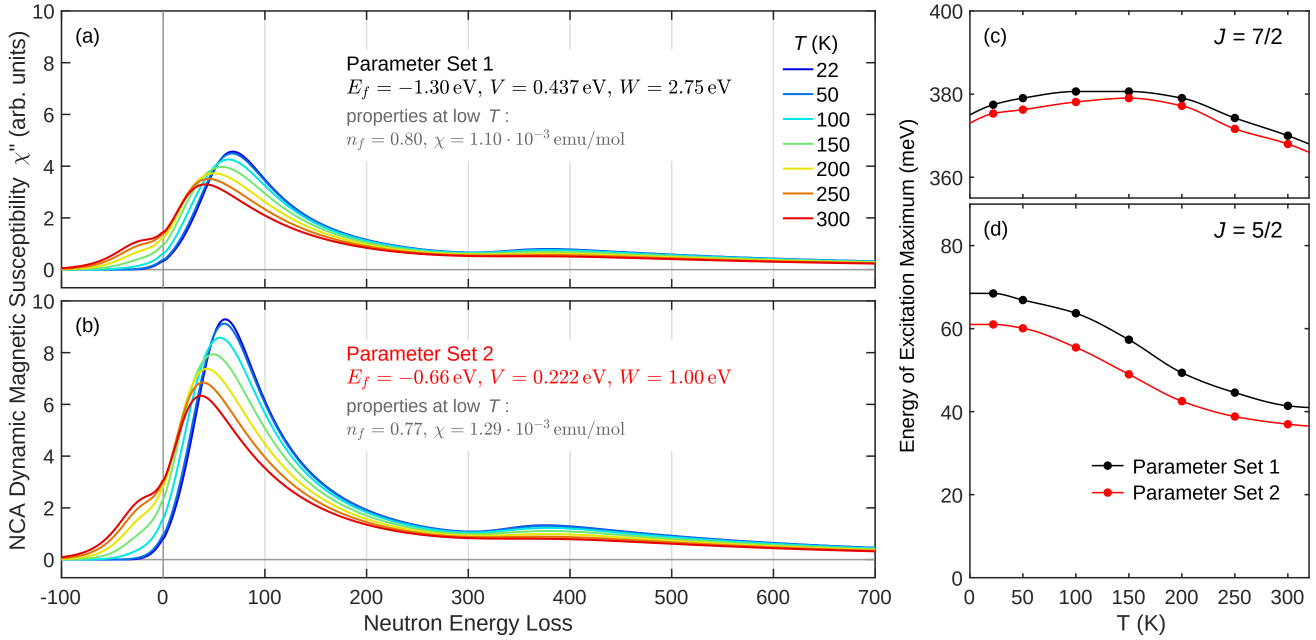


**Supplementary Figure 1: Dynamic magnetic susceptibility calculated for an Anderson impurity "toy model".** (a,b) NCA/AIM calculations of the momentum-averaged neutron magnetic scattering function $S(\omega) = [1 + n(\omega)]\frac{1}{\pi}\chi''(\omega)$, for a range of temperatures. The corresponding sets of model parameters $E_f$, $V$ and $W$ are indicated in each panel, along with the resulting values of $n_f$ and $\chi$ at $5\,K$. (c,d) Position of the corresponding maxima of $J = 5/2$ and $J = 7/2$ excitations. Lines are intended as a guide to the eye.

## Supplementary Note 2: Comparison of RIXS and DFT+DMFT calculations of $\chi''$

Goremychkin *et al.* have calculated the dynamic magnetic susceptibility of $CePd_3$ as the Lindhard susceptibility of a DFT+DMFT simulation, taking into account two-particle vertex corrections $\Gamma^{\mathrm{loc}}_{\mathrm{irr}}$ [5]. In Supplementary Figure 2, we present a comparison of these results with RIXS spectra obtained at similar momentum transfers and temperatures (cf. colored lines/data adopted from the Supplementary Materials of Ref. [5]). For reference, we also show the results of an earlier DFT+DMFT calculation by Sakai [6] (dashed line), as well as our AIM/NCA calculation, as discussed above (dot-dashed line).

The direct comparison between RIXS spectra and three computational models of $\chi''$ reveals some interesting similarities and differences. As in our simpler AIM/NCA model, $\chi''$ obtained by DFT+DMFT is dominated by excitations within the $J = 5/2$ ground state manifold and features only a weak broad signal at the spin-orbit energy. However, the DFT+DMFT spectra by Goremychkin *et al.* show little or no shift of the $J = 5/2$ excitation at low temperatures. Instead, an intermediate excitation emerges at around 150–200 meV. We have probed for this excitation using INS with an incident neutron energy of 400 meV [see purple markers in Supplementary Figure 3(b)] and confirmed its absence. Instead, the $J = 5/2 \to 5/2$ neutron excitations are in excellent agreement with RIXS. Even though the origin of the 150–200 meV feature in DFT+DMFT is unclear, it shares the dispersive trend of the $J = 5/2 \to 7/2$ excitation observed in RIXS.

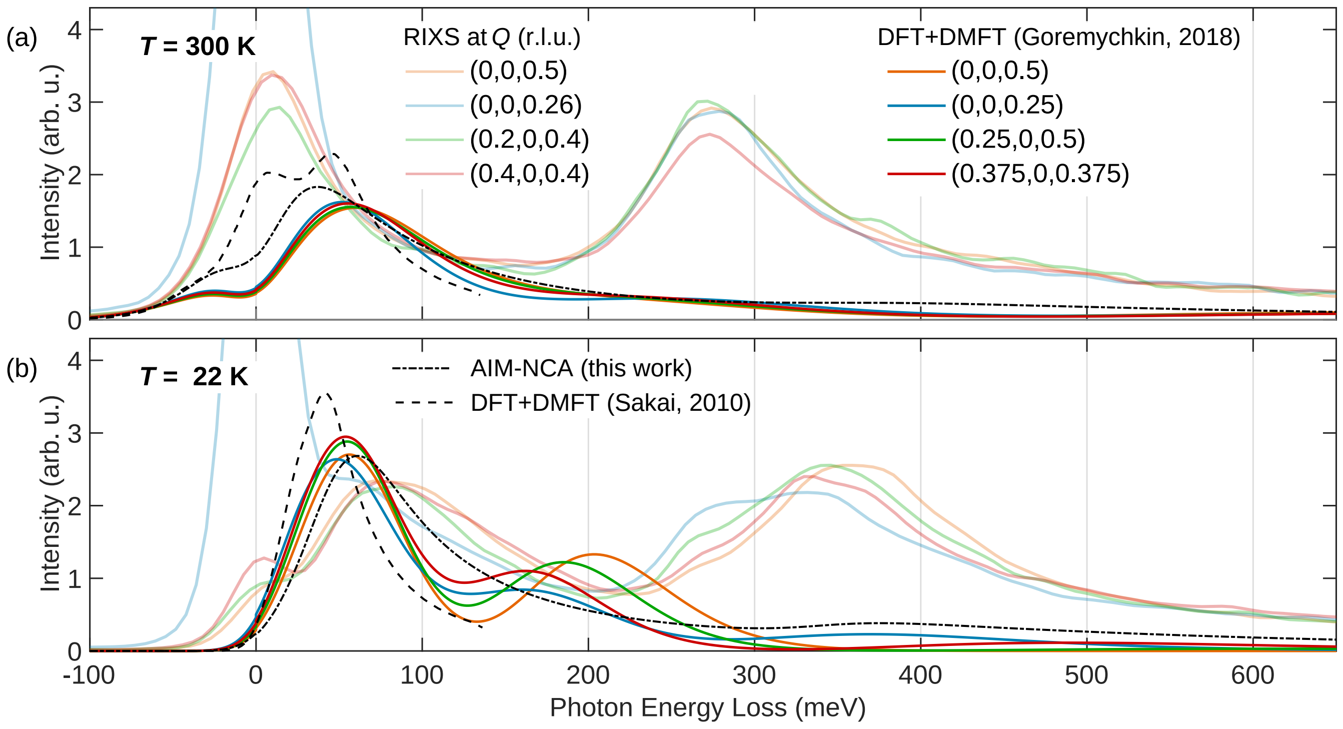


**Supplementary Figure 2: Comparison of RIXS spectra of $CePd_3$ with various calculations of its dynamic magnetic susceptibility, $\chi''(Q, \omega)$.** (a) At room temperature, and (b) at $T \ll T_{\mathrm{coh}}$. DFT+DMFT calculations are reproduced from Goremychkin *et al.* [5] (online materials), as well as from Sakai [6]. The results of our NCA/AIM "toy model" discussed in Supplementary Note 1 are also shown (Parameter Set 1). Lines drawn in corresponding colors indicate data measured/calculated at similar momentum transfer. Each set of calculations is arbitrarily scaled to the RIXS data, however with the same scale factor for high and low temperature calculations.

## Supplementary Note 3: Comparison of RIXS and inelastic neutron scattering

The fact that the RIXS excitations of $CePd_3$ appear in the crossed polarization channel suggests that the dynamic magnetic susceptibility $\chi''(Q,\omega)$, as measured by INS, may provide a useful comparison. We measured the polycrystalline-averaged dynamical correlation function $S(\omega) = [1+n(\omega)]\frac{1}{\pi}\chi''(\omega)$ [ $n(\omega)$: Bose occupation factor] on at ARCS (ORNL), which allows access to the 50–250 meV range. The spectrum is shown in Supplementary Figure 3(b) along with data reproduced from Fanelli *et al.* [3] (measurement at 7 K), Murani *et al.* [7] (10 K), and Murani *et al.* [8] (12 K). For reference, the RIXS spectra of Figure 3 of the manuscript are reproduced in Panel (a), and the ranges of these RIXS intensities are also indicated in Panel (b) by gray shaded margins. Where available, the spectra are presented in absolute units.

Remarkably, RIXS and INS are quantitatively consistent up to $\sim$ 200 meV, i.e., both in the energy position, lineshape, and in the relative intensity between low and high-temperature datasets [3]. On the other hand, RIXS and INS differ markedly at energy transfers above 200 meV. Due to the weakness of dipole transition matrix elements between the $J = 5/2$ and $J = 7/2$–like states, excitations across the spin-orbit gap are hardly measurable in INS. For reference, the red markers in Supplementary Figure 3(b) show results of the epicadmium (very high incident energy) INS study by Murani *et al.*, Ref [8]. After subtracting a $J = 5/2$ Lorentzian tail, the authors identified a weak spin-orbit feature, shown here enlarged by a factor 180.

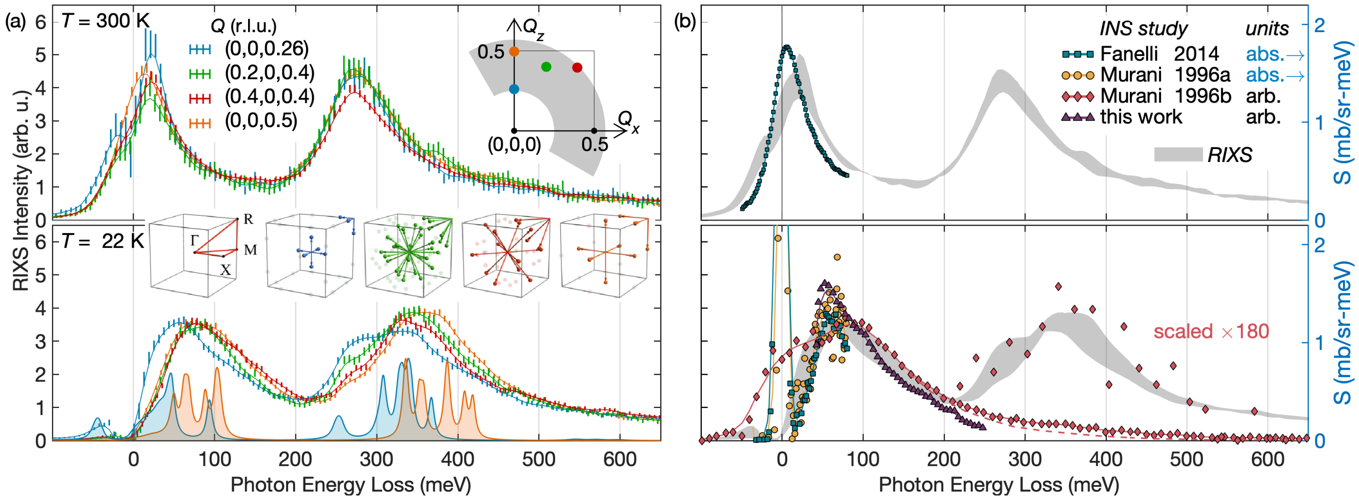


**Supplementary Figure 3: Emergent RIXS momentum-dependence and comparison with neutron scattering.** (a) RIXS spectra at four different momentum transfers $Q$, at 300 K (top) and 22 K (bottom). The positions of $Q$ are indicated in the reciprocal space map inset, where the $Q$ range accessible to $M_5$-edge RIXS is shaded gray. The lower inset illustrates the corresponding momentum transfers to quasiparticles in the Fermi surface pockets at Γ and R. For the extremal cases, $Q = (0,0,0.26)$ and $(0,0,0.5)$, the DFT+DMFT density of states summed over the relevant positions in the Brillouin zone are drawn as shaded peaks in corresponding colors. (b) Quantitative comparison of the RIXS spectra presented in Panel (a), here drawn as shaded margins, with polycrystalline-averaged neutron spectra of $CePd_3$ reported by Fanelli *et al.*, Ref. [3] and Murani *et al.*, Ref. [7, 8]. The neutron data marked *this work* has not been previously published. Datasets in which the neutron scattering function $S$ is available in absolute units (mb/sr-meV) are marked [abs.] in the legend (cf. right axis).

## Supplementary Note 4: DFT+DMFT AIM-RIXS calculation of the Kramers-Heisenberg term

### 4.1 Overview of the Method

For our computation of Ce $M_5$-edge RIXS spectra using the DFT+DMFT scheme, we proceed in several steps [9–15]. First, a standard DFT+DMFT calculation is performed for the experimental crystal structure of $CePd_3$, as follows. The DFT bands within the generalized gradient approximation (GGA) [16] for the exchange-correlation potential are obtained using the WIEN2K package [17], which implements the augmented plane wave and the local-orbital (APW+lo) method. Muffin-tin radii (RMT) of $2.5\,r_{\mathrm{Bohr}}$ are used for Ce and Pd atoms. The maximum modulus for the reciprocal vectors $K_{\mathrm{max}}$ was chosen such that $R_{\mathrm{MT}} \times K_{\mathrm{max}} = 7.0$. The Brillouin zone was sampled in a $20 \times 20 \times 20$ $k$-mesh. The spin-orbit coupling (SOC) is also taken into account in the GGA calculations.

Second, the DFT bands are projected onto a tight-binding model spanning Ce $4f$ and $5d$ bands, and Pd $4d$, $5s$, and $5p$ bands using the wien2wannier [18] and wannier90 [19] codes. This tight-binding model is augmented by the local electron-electron interaction $U$ within the Ce $4f$ shell. Following previous DFT+DMFT and spectroscopy studies for Ce compounds [5, 20], we employed $U = 6$ eV for the Hubbard parameter. The bare energy of the Ce 4f states is obtained from the GGA value by subtracting the double-counting correction $\mu_{\mathrm{dc}}$, which accounts for the Ce $4f$–$4f$ interaction already present in the GGA description. In the absence of a unique definition of $\mu_{\mathrm{dc}}$, we treat $\mu_{\mathrm{dc}}$ as a parameter, adjusted by comparison to experimental valence and inverse photoemission spectra [21, 22] (see below / Supplementary Figure 5).

Finally, to compute Ce $M_5$-edge RIXS and XAS spectra using the Kramers-Heisenberg formula [23] and Fermi's golden rule, we adopt the configuration-interaction (CI) solver [13, 15, 24]. This AIM solver uses the hybridization density $V^2(\varepsilon)$ obtained from DMFT. The AIM is augmented by the Ce $3d$ core states and the $3d$–$4f$ interaction present in the intermediate state of RIXS (corresponding to the final state of XAS). The core-hole potential $U_{fc}$ is set to 8.8 eV, and the higher Slater integrals obtained from atomic Hartree-Fock calculation [25] are scaled down to 80 % of their actual values to simulate the effect of intra-atomic configuration interaction from higher basis configurations neglected in the atomic calculation [25–27].

As the CI solver works directly in real frequencies, it allows to resolve fine spectral features in the spectra. However, it needs to approximate the continuum hybridization densities $V^2(\varepsilon)$ by a finite set of discrete bath levels. This CI method allows us to include more bath levels than in a standard exact diagonalization algorithm (see recent studies for $3d$ transition metal oxides [15, 24]). Nevertheless, for the case of a Ce $4f$ impurity with a large number of internal (local) degrees of freedom, the necessarily finite number of bath levels still poses a severe limitation.

### 4.2 Projection of bands onto a local basis

The Wannier functions $w(\mathbf{r})$ representing the Ce $4f$ states in the tight-binding model are summarized in Supplementary Figure 4. In projecting the Ce $4f$ bands, we adopt the $\Gamma_{6,7,8}$ basis, i.e. these Wannier functions are constructed directly as spinors (with different up and down components). The spin components of the Ce $4f$ Wannier functions are displayed in Supplementary Figure 4. The local Hamiltonian at the Ce site can be found in the table shown in this figure, where small off-diagonal matrix elements are allowed between the $\Gamma_7 - \Gamma_7'$ and $\Gamma_8 - \Gamma_8'$ states.

Since our Ce $4f$ Wannier functions are an irreducible representation of the cubic symmetry, the off-diagonal hybridization densities $V^2(i\omega_n)$ [discussed in Supplementary Note 4.4] are allowed only between the states belonging to the same (two $\Gamma_7$ or two $\Gamma_8$) representations. Given that the two $\Gamma_7$ (or $\Gamma_8$) states are well split by the spin-orbit coupling, the resulting off-diagonal hybridization densities are small [cf. Supplementary Figure 6(c)].

To model the electron-electron interaction in the AIM calculation, these small off-diagonal hybridization densities are neglected, and only the isotropic Hubbard term $U$ is taken into account. Using this computational setup, the CT-QMC simulation can access low temperatures, free from the sign problem.

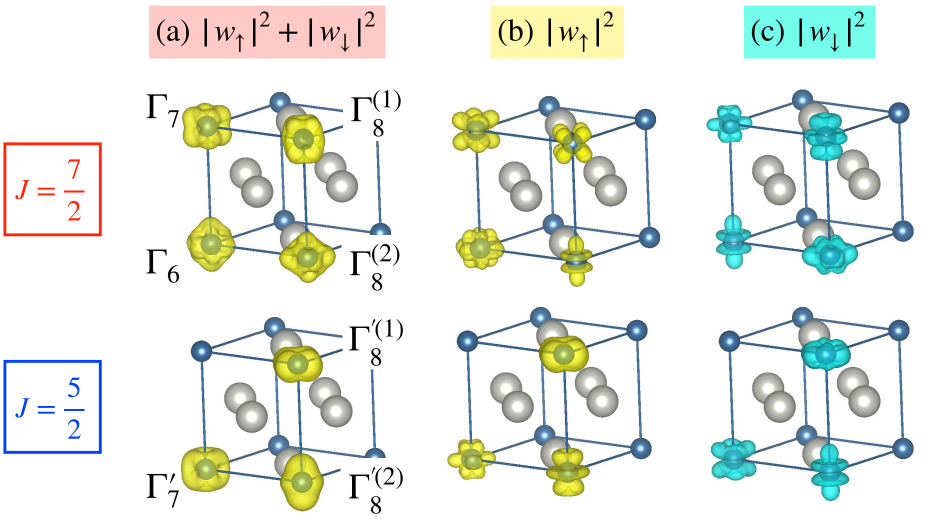


$H_{\text{loc@Ce site}} =$ ($J = \frac{7}{2}$: $\Gamma_6$, $\Gamma_7$, $\Gamma_8$ blocks; $J = \frac{5}{2}$: $\Gamma_7'$, $\Gamma_8'$ blocks)

| $\Gamma_6(2)$ | | $\Gamma_7(2)$ | | $\Gamma_8(4)$ | | | | $\Gamma_7'(2)$ | | $\Gamma_8'(4)$ | | | |
|---|---|---|---|---|---|---|---|---|---|---|---|---|---|
| 0.4881 | 0 | 0 | | 0 | | | | 0 | | 0 | | | |
| | 0.4881 | | | | | | | | | | | | |
| | | 0.5218 | 0 | 0 | | | | 0.0994 | 0 | 0 | | | |
| | | | 0.5218 | | | | | 0 | -0.0994 | | | | |
| | | | | 0.5846 | 0 | 0 | 0 | 0 | | -0.0714 | 0 | 0 | 0 |
| | | | | | 0.5846 | 0 | 0 | | | 0 | 0.0714 | 0 | 0 |
| | | | | | | 0.5846 | 0 | | | 0 | 0 | -0.0714 | 0 |
| | | | | | | | 0.5846 | | | 0 | 0 | 0 | 0.0714 |
| | | | | | | | | 0.2102 | 0 | 0 | | | |
| | | | | | | | | | 0.2102 | | | | |
| | | | | | | | | | | 0.2033 | 0 | 0 | 0 |
| | | | | | | | | | | | 0.2033 | 0 | 0 |
| | | | | | | | | | | | | 0.2033 | 0 |
| | | | | | | | | | | | | | 0.2033 |

**Supplementary Figure 4: Projection of the calculated $f$-electronic structure onto a basis of Wannier orbitals**. (a) The density of the Ce 4f Wannier functions ($|w_\uparrow|^2 + |w_\downarrow|^2$), (b) of the up-spin components ($|w_\uparrow|^2$), and (c) of the down-spin components ($|w_\downarrow|^2$) of the wannier functions. The local Hamiltonian $H_{\rm loc}$ (the upper triangle part) at the Ce site is shown in the table.

### 4.3 The double counting parameter $\mu_{\rm dc}$

As stated above, the bare energy of the Ce 4f states is obtained from the GGA value by subtracting the double-counting correction $\mu_{\rm dc}$, which accounts for the Ce $4f$–$4f$ interaction that is already present in the GGA description. In practice, $\mu_{\rm dc}$ renormalizes the energy splitting between the Ce $4f$ states and Pd $4d$ (and other) bands, which is illustrated in Supplementary Figure 5. As a consequence, the position of the $4f^2$ peak (at around 5 eV in the experimental data) is sensitive to $\mu_{\rm dc}$, despite a small change in the Ce $4f$ occupation ($N_{4f}$ in the figure). This allows us to fix $\mu_{\rm dc} \approx -2.25$ eV.

Aside from these valence spectra (Supplementary Figure 5), the obtained Ce $4f$ self-energies $\Sigma(\varepsilon)$ are also highly sensitive to $\mu_{\rm dc}$, as discussed below in the context of Supplementary Figure 6.

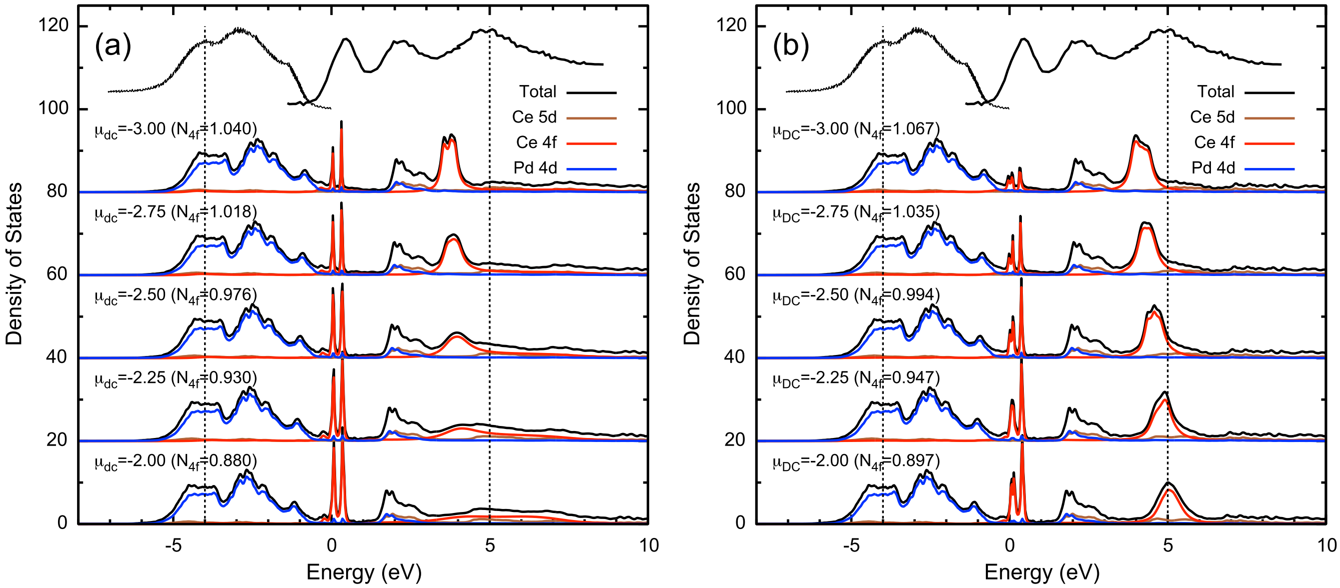


**Supplementary Figure 5: The double-counting ($\mu_{\rm dc}$) dependence of the valence spectra, as calculated by the DFT+DMFT method.** (a) At low temperatures (116 K) and (b) at high temperatures (400 K). The experimental photoemission and inverse photoemission spectra are taken from Refs. [21, 22].

### 4.4 Self energies $\Sigma(\varepsilon)$

In DMFT, the electron dynamics on the Ce sites is described by the Anderson impurity model (AIM) [9, 10]. The exchange of the electrons between a Ce site and the rest of the crystal (represented by non-interacting auxiliary bath states) is described by the hybridization density $V^2(i\omega_n)$ that is determined self-consistently within DMFT. We use a strong-coupling continuous-time quantum Monte Carlo (CT-QMC) impurity solver [28–31] to compute the self-energies $\Sigma(i\omega_n)$ from the AIM.

Aside from the valence spectra (Supplementary Figure 5), the Ce $4f$ self-energies $\Sigma(\varepsilon)$ also show a strong $\mu_{\rm dc}$ dependence, see Supplementary Figure 6. This relates with the physics of a highly asymmetric Anderson impurity model, where the $4f^2$ state (upper Hubbard band) lies at around 5 eV, while the $4f^0$ state (lower Hubbard band) is close to the chemical potential. In terms of ionic configurations, the $f^0$ state is rather close to the $f^1$ ground state, and thus even a small shift of the bare $4f$ levels (by the double-counting correction $\mu_{\rm dc}$) yields a large change of $E(f^1) - E(f^0)$. This also affects the Kondo temperature. Consequently, the thermal evolution of both the low-energy Ce $4f$ spectra (Supplementary Figure 5) and the self energy (Supplementary Figure 6) depend on $\mu_{\rm dc}$.

Given this $\mu_{\rm dc}$-dependence, we do not implement the charge self-consistency (updating the DFT charge) in our DFT+DMFT calculation. Instead we adjust $\mu_{\rm dc}$ as a key parameter to achieve consistency with experimental results. This includes previously reported (direct and inverse) photoemission spectra (Supplementary Figure 5), as well as the spectroscopic results (ARPES, $M$-edge XAS, RIXS) reported in the manuscript.

After reaching DMFT self-consistency, we analytically continue $\Sigma(i\omega_n)$ with the maximum entropy method [32, 33] to $\Sigma(\varepsilon)$ in real frequencies $\varepsilon$. $\Sigma(\varepsilon)$ is then used to calculate the one-particle spectral densities $A(\varepsilon)$ and hybridization densities $V^2(\varepsilon)$ at real frequencies, as shown in Supplementary Figure 6(c).

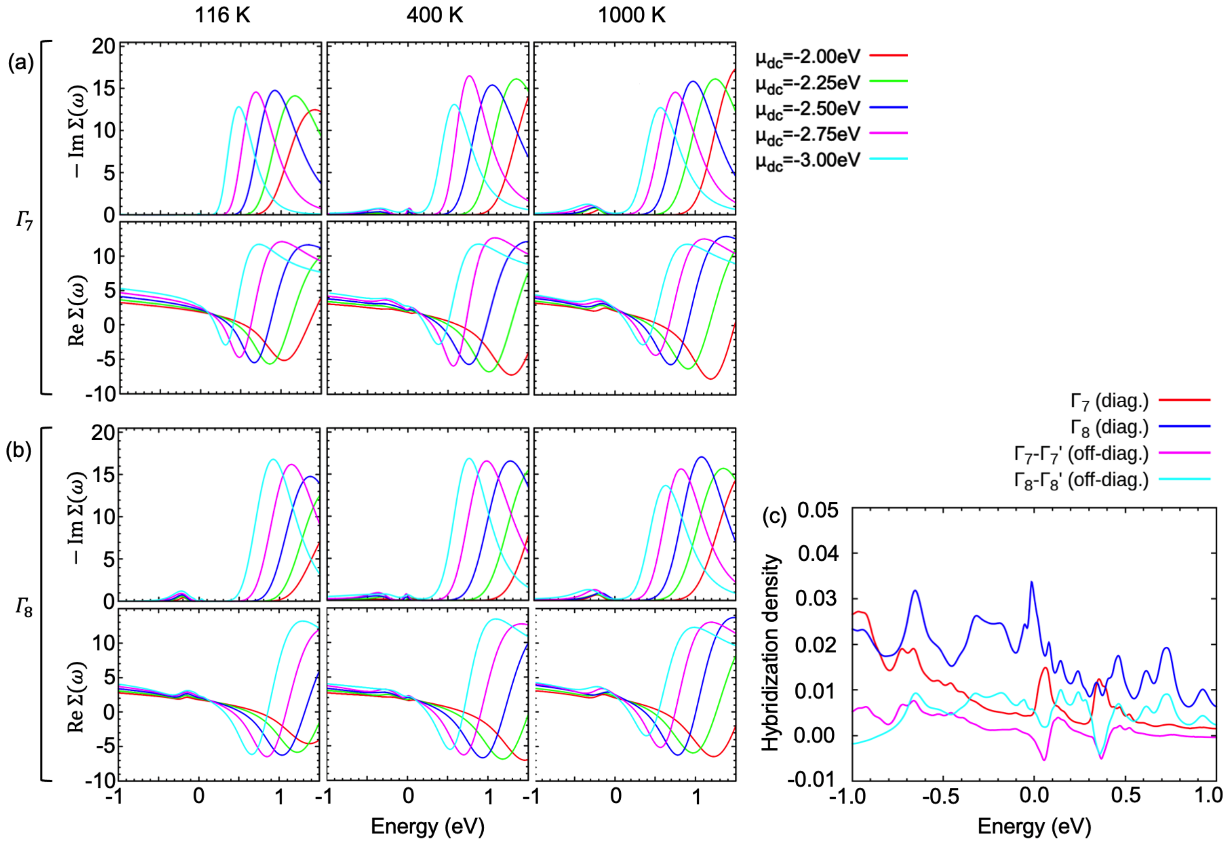


**Supplementary Figure 6: Real and imaginary parts of the self energy in $CePd_3$, calculated for different double counting parameters $\mu_{\rm dc}$, at 116 K, 400 K, and 1000 K.** (a) For $\Gamma_7$ orbitals and (b) for $\Gamma_8$ orbitals. (c) Hybridization density for $\Gamma_7$ and $\Gamma_8$, as well as for off-diagonal terms.

### 4.5 Discretization of hybridization densities

As noted above, the AIM solver works directly in real frequencies. This allows to resolve fine spectral features in the spectra, but requires to approximate the continuum hybridization densities $V^2(\varepsilon)$ by discrete levels. In the present study, we modeled $V^2(\varepsilon)$ by 20 bath levels in the discretization scheme $[-5 : 1 : -1, -0.08 : 0.02 : 0, 0.25 : 0.25 : 2.5]$ eV, as illustrated below.

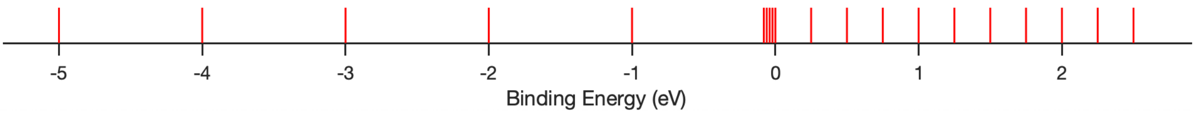


The CI implementation of the present discrete bath gives an accurate $4f$ occupation $N_{4f}$ (the deviation from the numerically-exact one estimated by CT-QMC is less than 5%) for the used $\mu_{\rm dc}$ value ($-2.25$ eV) and simultaneously reproduces the energy of the $J = 5/2 \rightarrow 5/2$ transitions ($\sim 70$ meV, see the main text). In testing various discretization schemes of $V^2(\varepsilon)$, we found that only those with an accurate $N_{4f}$ value also give a good match in the position of the $J = 5/2 \rightarrow 5/2$ peak. At high temperatures, low-energy RIXS features are basically atomic excitations involving the $J = 7/2$ and $J = 5/2$ manifolds, i.e., the given Ce site and the rest of the crystal are effectively decoupled from each other. Thus the details in the bath discretization do not affect the low-energy RIXS features.

## Supplementary Note 5: RIXS incident energy dependence and Pd $4d$ bands

Supplementary Figure 7 shows the variation of RIXS spectra of $CePd_3$ in the vicinity of the Ce $M_5$ absorption edge. The measurements were obtained at room temperature, by exciting the sample with x-rays linearly polarized in the scattering plane, and with polarization-resolution of the scattered beam ($\pi\pi'$+$\pi\sigma'$ polarisation channels). The data allows distinguishing a broad fluorescence-like signal with energy-transfers proportional to the excitation energy ($E \propto \hbar\omega_{\rm in}$) from the Raman-like ($E$ =const.) interband excitations. The right panel shows a detailed view of low energy transfers.

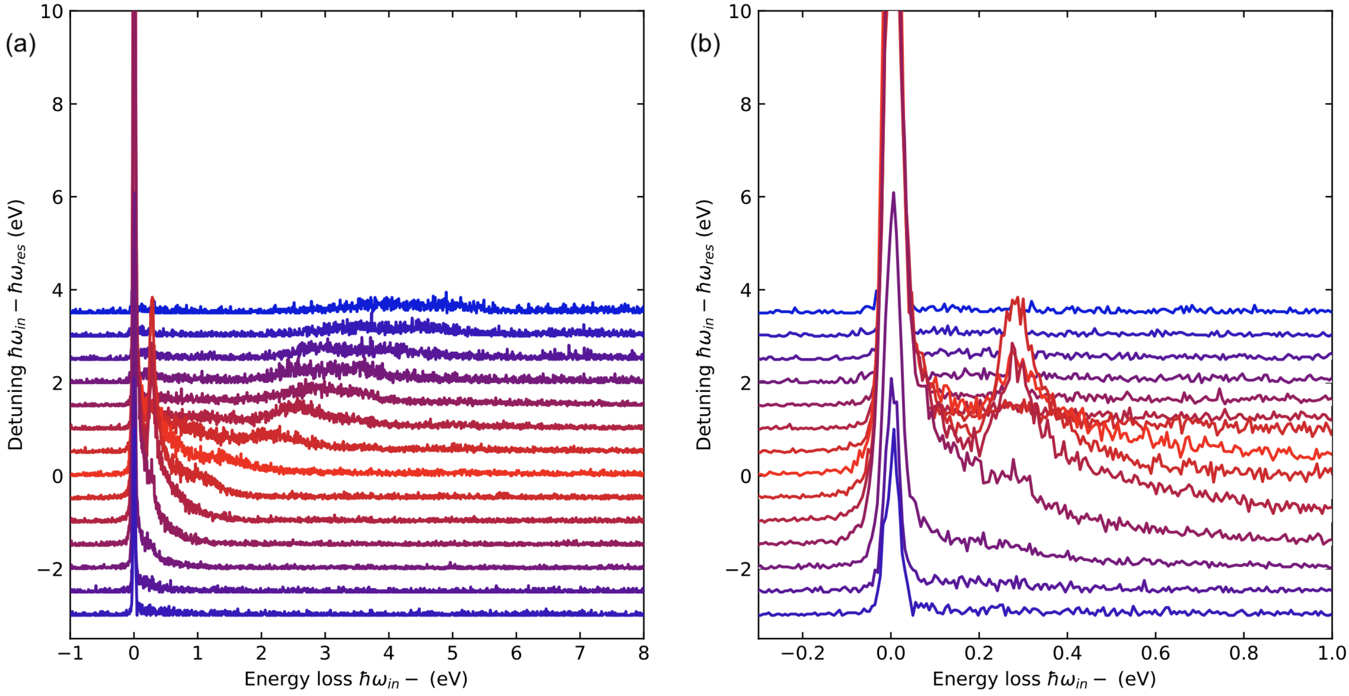


**Supplementary Figure 7: Variation of the RIXS response of $CePd_3$ with the incident photon energy.** (a) Waterfall plot illustrating the energy dependence in a wide region around the Ce $M_5$ edge. The spectra were obtained at room temperature, in specular scattering geometry, at a scattering angle of $2\theta = 150$, with incident x-rays linearly polarised in the scattering plane ($\pi\sigma'$+$\pi\pi'$). (b) Detailed view of these spectra at low energy transfer.

The fluorescence-like signal is due to excitations of quasiparticles into the highly itinerant conduction band. These Pd $4d$ states are illustrated in the extended DFT+DMFT band structure shown in Supplementary Figure 8.

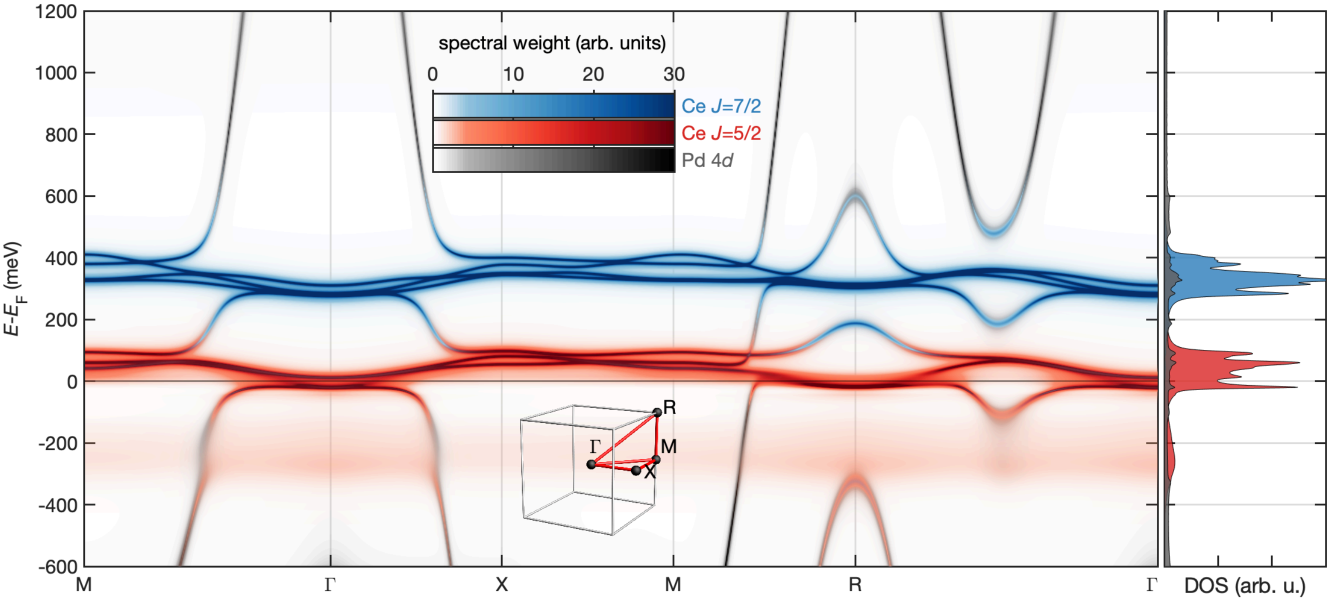


**Supplementary Figure 8: Extended view of the DFT+DMFT bands at 116 K.** The same calculation is shown as in Figure 1(f) of the manuscript, but over a broader energy range and here including the Pd $4d$ bands. The side panel shows the Ce $J = 7/2$, $J = 5/2$, and Pd $4d$ spectral weight summed along the path shown in the main panel.

## Supplementary Note 6: RIXS incident energy and polarization dependence

We combine RIXS polarization-analysis and photon-energy ($\omega_{\text{in}}$) dependence to separate the spin-orbit channels of the interband excitations and assign their character. As shown in Supplementary Figure 9(a), at $\omega_{\text{in}} = \omega_{\text{res}} - 1.5\,\text{eV}$, the incident energy does not suffice to access intermediate states in which Ce $f^1$ states have been excited to the $J = 7/2$ state. At $\omega_{\text{in}} = \omega_{\text{res}} - 0.5\,\text{eV}$, as shown in Panel (b), these excitations are strongly enhanced. The polarization analysis of the scattered beam reveals that they appear mostly in the crossed ($\sigma\pi'$) channel. This is further emphasized by subtracting the former from the latter spectra, see Panel (c), middle. The spectral weight attributable to spin-orbit excitations can thus be isolated. In turn, subtracting this signal from the $\omega_{\text{res}} - 0.5\,\text{eV}$ data separates excitations within the ground state manifold, see Panel (c), bottom. Overall, both transitions appear with similar spectral weight in the crossed polarization channel. The quasielastic Thompson scattering, as well as a sloping background signal appear in the parallel ($\sigma\sigma'$) channel.

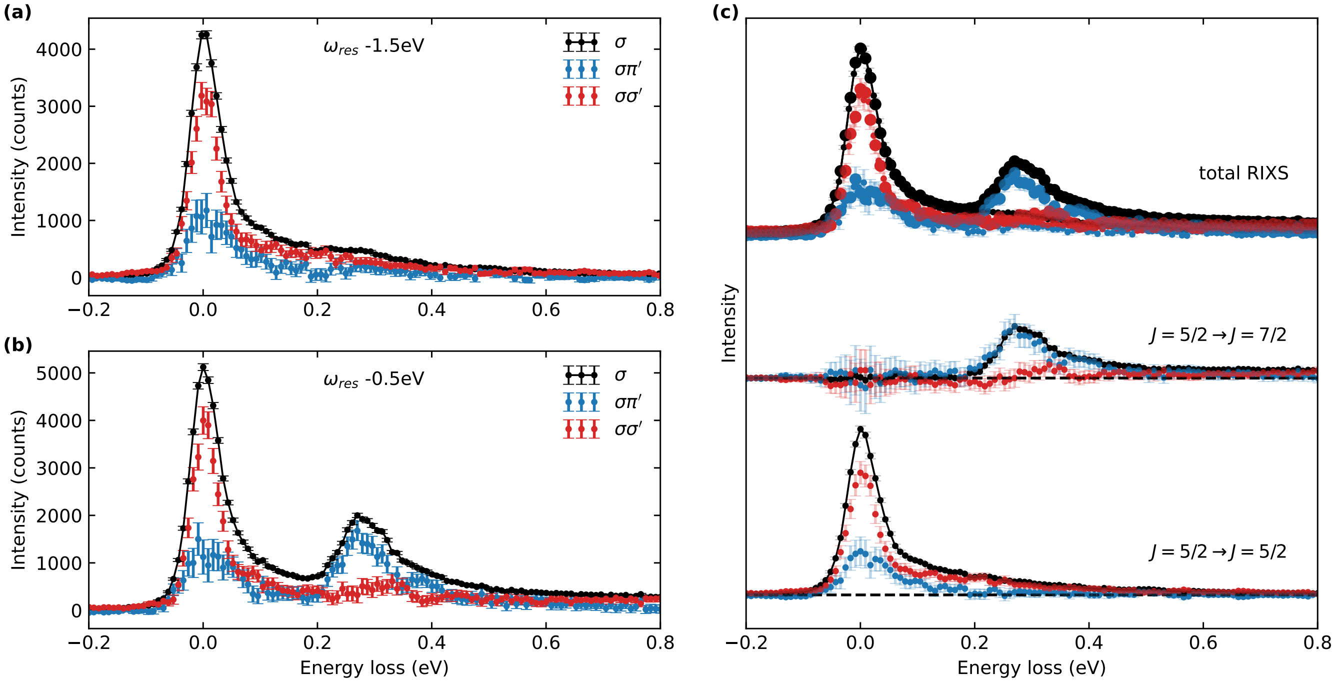


**Supplementary Figure 9: Polarization and energy dependence of the $M_5$ RIXS response of $CePd_3$.** (a) Spectra without polarization analysis, and in the separated $\sigma\pi'$ and $\sigma\sigma'$ channels, at room temperature, 1.5 eV below the resonance (≈880.7 eV). (b) The corresponding spectra obtained 0.5 eV below resonance (≈881.7 eV). (c) Top: Reproduction of the spectra as show in panels (a,b). Middle and bottom: Separation of the spin orbit channels ($J = 5/2 \rightarrow 5/2$, vs. $5/2 \rightarrow 7/2$), by subtraction of the spectra shown in Panels (a,b).

**Supplementary Note 7: Ce M-edge x-ray absorption**

The M-edge x-ray absorption spectroscopy (XAS) characteristics observed in this study are consistent with earlier fluorescence-yield measurements [34] and resemble those of other strongly valence fluctuating Ce materials [35].

In Supplementary Figure 10, we compare Ce $M$ edge x-ray absorption spectra of $CePd_3$ obtained at the RIXS instrument [in total electron yield (TEY) mode, as shown in Figure 2(d) of the manuscript] with those obtained in partial-fluorescence-yield (PFY) mode at the MaRES endstation of BL29/BOREAS (ALBA). During the RIXS experiment, TEY-XAS spectra were measured as the drain current from the sample surface (at 22 K). The photon energy range shown here covers both Ce $M_5$ ($3d_{5/2} \rightarrow 4f_{7/2}$, 884 eV) and $M_4$ ($3d_{3/2} \rightarrow 4f_{5/2}$, 902 eV) absorption edges, split by the spin-orbit coupling of the $3d^9$ core-hole. These TEY-XAS spectra did not vary with the incident angle of the beam, which indicates that the measurement is not strongly affected by surface contamination or reconstruction. The intricate lineshapes reflect the impact of the core-hole potential on the configuration of the largely unoccupied $4f$ manifold, corresponding to the available intermediate states of the RIXS process. The photon energy $h\nu$ = 882.2 eV chosen in this study corresponds approximately to the center of the $M_5$ XAS double-peak in trivalent Ce compounds [34, 36, 37].

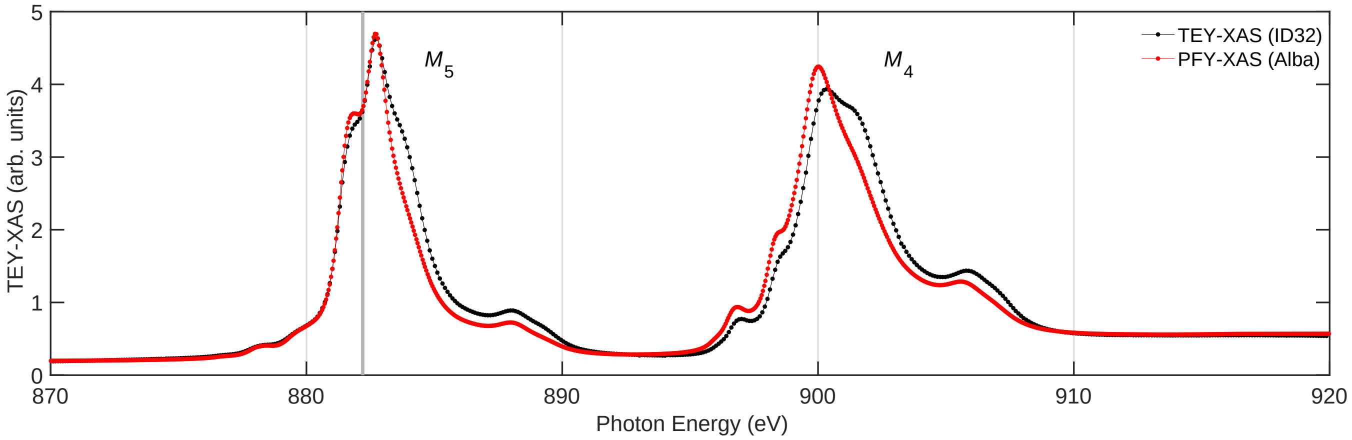


**Supplementary Figure 10: Cerium $M$ edge x-ray absorption spectra.** Data recorded in total electron yield mode at the RIXS instrument (ID32) is compared with partial fluorescence mode data obtained at BL29/BOREAS (ALBA). The resonance energy at which the RIXS spectra shown in the manuscript were obtained (882.2 eV), is marked by a gray line.

## Supplementary Note 8: Overview of RIXS spectra and fits

Below we provide a complete overview of the temperature-dependent Ce $M_5$ RIXS data discussed in the manuscript. A realistic model of these spectra, taking into account the dispersive $f$-band structure, is currently not feasible. The phenomenological analysis presented in Supplementary Figs. 11 and 12 is therefore not intended as an unequivocal interpretation. For instance, to allow convergence, peak positions have to be fixed in the changeover temperature range around $T_{\mathrm{coh}}$. It is therefore not meaningful to infer the exact positions of individual Lorentzian peaks. The purpose of this analysis is merely to illustrate the changeover between two lineshapes, which is evident even in the raw data.

The spectra at momentum-transfers $\mathbf{Q} = (0.395, 0, 0.395)$ and $\mathbf{Q} = (0.495, 0, 0)$ were each tracked in the temperature range of 22 K – 300 K, and are shown in Supplementary Figs. 11 and 12, respectively:

- Panels labeled (a) show the raw data at energy transfers between −100 and 700 meV. The (quasi-)elastic scattering is shaded gray. In the 22 K spectra [see panels (7a)], this contribution can be distinguished as a resolution-limited peak resembling the instrumental resolution function (i.e., a Gaussian distribution of FWHM 32 meV). We attribute this contribution to diffuse elastic scattering (e.g., due to minor bulk and surface structural defects) and hence assume that it is, as inferred from the 22 K spectra, independent of temperature. Up to four Lorentzian peaks, used as a phenomenological model, are indicated by colored lineshapes. The corresponding fits include a scale parameter $c$, the broadening $\Gamma$, and the resonance energy $E_0$,

$$L(E) = c\,E\,\frac{\Gamma/(2\pi)}{\Gamma^2 + (|E| - E_0)^2} \quad .$$

  Note that the maximum of this function lies at $E_{\mathrm{max}} = \sqrt{E_0^2 + \Gamma^2}$. This lineshape has proven to be a good approximation of the dynamic magnetic susceptibility calculated in NCA/AIM [38, 39] and thus provides an adequate model of the momentum-averaged neutron spectra of many Ce-intermetallics. The Principle of Detailed Balance was applied to these lineshapes to obtain the corresponding anti-Stokes spectral weight without further variables. A $\delta(E)$-like function was used to model the (quasi-)elastic scattering. The sum of these features was convoluted with the experimental resolution function.

- Panels (b) illustrate the same data after subtraction of the (quasi-)elastic scattering and photon-energy–gain intensity. The signal assigned to excitations within the $J = 5/2$ manifold and into the $J = 7/2$ states is indicated by red and blue linehshapes, respectively. The NCA/AIM calculations of $\chi''$ [Parameter Set 1, as shown in Supplementary Figure 1(a)] are superimposed as black dashed lines.

- Panels (c) show detail views of the very-low energy-transfer regime (−100–50 meV), with an emphasis on photon-energy–gain (anti-Stokes) excitations. The observation of this effect had not been possible with x-ray spectrometers of the previous generation (with energy resolution d$E > k_{\mathrm{B}}T$). The shaded areas indicate separately the $J = 5/2$ Lorentzian lineshape and the corresponding anti-Stokes intensity, obtained without further parameters by the Principle of Detailed Balance.

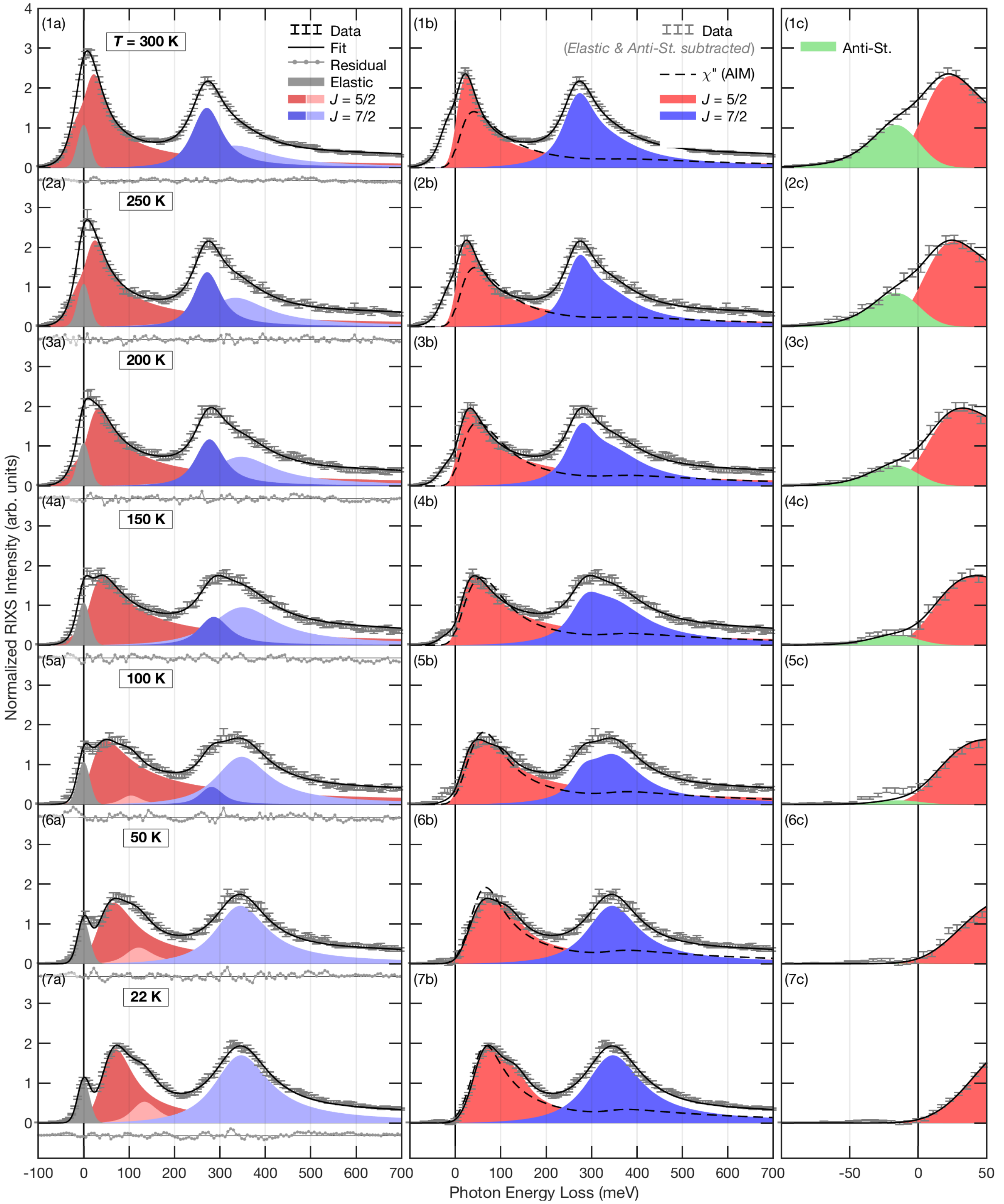


**Supplementary Figure 11: Overview of $CePd_3$ RIXS spectra measured at momentum transfer $\mathbf{Q} = (0.4, 0, 0.4)$, illustrating the thermal variation in the range of 22 K – 300 K.** (1a)–(7a) Raw data and phenomenological fits. Separate Lorentzian lineshapes are indicated by colored lineshapes and the temperature-independent (quasi-)elastic Gaussian peak (32 meV FWHM) is shaded gray. (1b)–(7b) Corresponding view of the spectra after subtraction of the (quasi-)elastic scattering. The NCA/AIM calculations of $\chi''(\omega)$ are shown as back dashed lines. (1c)–(7c) Detail view of the photon-energy–loss regime, emphasizing the evolution of anti-Stokes scattering at high temperatures. The seperated (anti-) Stokes contributions to the low-energy RIXS intensity are indicated as shaded areas.

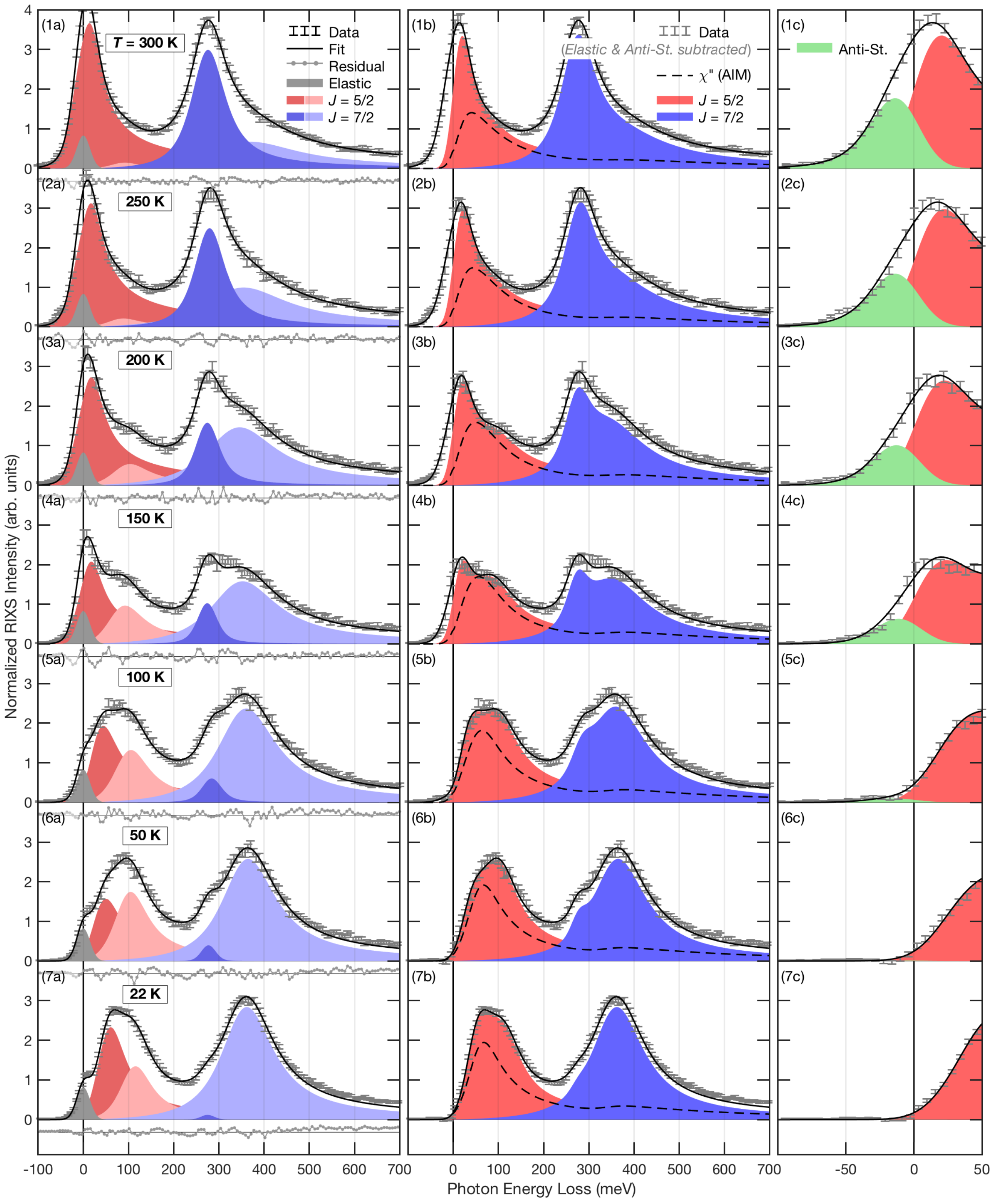


**Supplementary Figure 12: Overview of $CePd_3$ RIXS spectra measured at a momentum transfer of $\mathbf{Q} = (0.5, 0, 0)$, illustrating the thermal variation in the range of 22 K − 300 K.** The data is presented in analogy to Supplementary Figure 11.

[1] Bickers, N. E. Review of techniques in the large-N expansion for dilute magnetic alloys. *Rev. Mod. Phys.* **59**, 845 (1987).

[2] Lawrence, J. M. *et al.* Slow crossover in Yb$X$Cu$_4$ ($X$ = Ag, Cd, In, Mg, Tl, Zn) intermediate-valence compounds. *Physical Review B* **63**, 054427 (2001). https://link.aps.org/doi/10.1103/PhysRevB.63.054427.

[3] Fanelli, V. R. *et al.* Q-dependence of the spin fluctuations in the intermediate valence compound CePd$_3$. *J. Phys.: Condens. Matter* **26**, 225602 (2014).

[4] Lawrence, J. M. Intermediate Valence Metals. *Modern Physics Letters B* **22**, 1273–1295 (2008). http://www.worldscientific.com/doi/abs/10.1142/S0217984908016042.

[5] Goremychkin, E. A. *et al.* Coherent band excitations in CePd$_3$ : A comparison of neutron scattering and ab initio theory. *Science* **359**, 186–191 (2018). 1611.01149.

[6] Sakai, O. Band Calculations for Ce Compounds with AuCu$_3$-type Crystal Structure on the basis of Dynamical Mean Field Theory: I. CePd3 and CeRh3. *Journal of the Physical Society of Japan* **79**, 114701 (2010). URL `http://journals.jps.jp/doi/abs/10.1143/JPSJ.79.114701`. 1009.0327.

[7] Murani, A. P., Severing, A. & Marshall, W. G. Paramagnetic spectral response of CePd$_3$: A comparative investigation of neutron inelastic scattering from single-crystal and polycrystalline samples. *Phys. Rev. B* **53**, 2641–2650 (1996). https://link.aps.org/doi/10.1103/PhysRevB.53.2641.

[8] Murani, A. P., Raphel, R., Bowden, Z. A. & Eccleston, R. S. Kondo resonance energies in CePd$_3$. *Phys. Rev. B* **53**, 8188–8191 (1996). https://link.aps.org/doi/10.1103/PhysRevB.53.8188.

[9] Georges, A., Kotliar, G., Krauth, W. & Rozenberg, M. J. Dynamical mean-field theory of strongly correlated fermion systems and the limit of infinite dimensions. *Rev. Mod. Phys.* **68**, 13–125 (1996). URL `http://link.aps.org/doi/10.1103/RevModPhys.68.13`.

[10] Kotliar, G. *et al.* Electronic structure calculations with dynamical mean-field theory. *Rev. Mod. Phys.* **78**, 865–951 (2006). URL `http://link.aps.org/doi/10.1103/RevModPhys.78.865`.

[11] Kuneš, J. *et al.* Dynamical mean-field approach to materials with strong electronic correlations. *Eur. Phys. J. Spec. Top.* **180**, 5–28 (2009). URL `http://dx.doi.org/10.1140/epjst/e2010-01209-0`.

[12] Kuneš, J. *et al.* LDA+DMFT approach to ordering phenomena and the structural stability of correlated materials. *The European Physical Journal Special Topics* **226**, 2641–2675 (2017). URL `https://doi.org/10.1140/epjst/e2017-70054-6`.

[13] Hariki, A., Uozumi, T. & Kuneš, J. LDA+DMFT approach to core-level spectroscopy: Application to 3$d$ transition metal compounds. *Phys. Rev. B* **96**, 045111 (2017). URL `https://link.aps.org/doi/10.1103/PhysRevB.96.045111`.

[14] Hariki, A., Winder, M. & Kuneš, J. Continuum charge excitations in high-valence transition-metal oxides revealed by resonant inelastic x-ray scattering. *Phys. Rev. Lett.* **121**, 126403 (2018). https://link.aps.org/doi/10.1103/PhysRevLett.121.126403.

[15] Hariki, A., Winder, M., Uozumi, T. & Kuneš, J. LDA+DMFT approach to resonant inelastic x-ray scattering in correlated materials. *Phys. Rev. B* **101**, 115130 (2020). https://link.aps.org/doi/10.1103/PhysRevB.101.115130.

[16] Perdew, J. P., Burke, K. & Ernzerhof, M. Generalized gradient approximation made simple. *Phys. Rev. Lett.* **77**, 3865–3868 (1996). URL `https://link.aps.org/doi/10.1103/PhysRevLett.77.3865`.

[17] Blaha, P., Schwarz, K., Madsen, G., Kvasnicka, D. & Luitz, J. *WIEN2k, An Augmented Plane Wave + Local Orbitals Program for Calculating Crystal Properties* (Karlheinz Schwarz, Techn. Universitat Wien, Austria, 2001, ISBN 3-9501031-1-2).

[18] Kuneš, J. *et al.* Wien2wannier: From linearized augmented plane waves to maximally localized wannier functions. *Comput. Phys. Commun.* **181**, 1888 – 1895 (2010). URL `http://www.sciencedirect.com/science/article/pii/S0010465510002948`.

[19] Mostofi, A. A. *et al.* An updated version of wannier90: A tool for obtaining maximally-localised wannier functions. *Comput. Phys. Commun.* **185**, 2309 – 2310 (2014). URL `http://www.sciencedirect.com/science/article/pii/S001046551400157X`.

[20] Sakai, O., Shimizu, Y. & Kaneta, Y. Band calculation for Ce-compounds on the basis of dynamical mean field theory. *J. Phys. Soc. Jpn.* **74**, 2517–2529 (2005). URL `https://doi.org/10.1143/JPSJ.74.2517`. https://doi.org/10.1143/JPSJ.74.2517.

[21] Malterre, D., Grioni, M., Weibel, P., Dardel, B. & Baer, Y. Evidence of a Kondo scale from the temperature dependence of inverse photoemission spectroscopy of CePd$_3$. *Phys. Rev. Lett.* **68**, 2656–2659 (1992). URL `https://link.aps.org/doi/10.1103/PhysRevLett.68.2656`.

[22] Souma, S., Kumigashira, H., Ito, T., Takahashi, T. & Kasaya, M. Ultrahigh-resolution photoemission study of CePd$_3$: absence of Kondo insulator gap. *J. Electron Spectrosc.* **114-116**, 735 – 740 (2001). Proceeding of the Eight International Conference on Electronic Spectroscopy and Structure,, http://www.sciencedirect.com/science/article/pii/S0368204800003868.

[23] Kramers, H. A. & Heisenberg, W. Über die Streuung von Strahlung durch Atome. *Z. Phys.* **31**, 681–708 (1925). URL `https://doi.org/10.1007/BF02980624`.

[24] Winder, M., Hariki, A. & Kuneš, J. X-ray spectroscopy of the rare-earth nickelate LuNiO$_3$: DFT+DMFT study. *Phys. Rev. B* **102**, 085155 (2020). URL `https://link.aps.org/doi/10.1103/PhysRevB.102.085155`.

[25] Cowan, R. D. *The Theory of Atomic Structure and Spectra (Los Alamos Series in Basic and Applied Sciences)* (University of California Press, 1981).

[26] Sugar, J. Potential-barrier effects in photoabsorption. ii. interpretation of photoabsorption resonances in lanthanide metals at the 4$d$-electron threshold. *Phys. Rev. B* **5**, 1785–1792 (1972). URL `https://link.aps.org/doi/10.1103/PhysRevB.5.1785`.

[27] de Groot, F. & Kotani, A. *Core Level Spectroscopy of Solids* (CRC Press, Boca Raton, FL, 2014).

[28] Werner, P., Comanac, A., de' Medici, L., Troyer, M. & Millis, A. J. Continuous-time solver for quantum impurity models. *Phys. Rev. Lett.* **97**, 076405 (2006). http://link.aps.org/doi/10.1103/PhysRevLett.97.076405.

[29] Boehnke, L., Hafermann, H., Ferrero, M., Lechermann, F. & Parcollet, O. Orthogonal polynomial representation of imaginary-time Green's functions. *Phys. Rev. B* **84**, 075145 (2011). URL `http://link.aps.org/doi/10.1103/PhysRevB.84.075145`.

[30] Hafermann, H., Patton, K. R. & Werner, P. Improved estimators for the self-energy and vertex function in hybridization-expansion continuous-time quantum Monte Carlo simulations. *Phys. Rev. B* **85**, 205106 (2012). URL `http://link.aps.org/doi/10.1103/PhysRevB.85.205106`.

[31] Hariki, A., Yamanaka, A. & Uozumi, T. Theory of spin-state selective nonlocal screening in Co $2p$ x-ray photoemission spectrum of $LaCoO_3$. *J. Phys. Soc. Jpn.* **84**, 073706 (2015). http://dx.doi.org/10.7566/JPSJ.84.073706.

[32] Jarrell, M. & Gubernatis, J. Bayesian inference and the analytic continuation of imaginary-time quantum Monte Carlo data. *Phys. Rep.* **269**, 133 – 195 (1996). URL `http://www.sciencedirect.com/science/article/pii/0370157395000747`.

[33] Wang, X., Gull, E., de' Medici, L., Capone, M. & Millis, A. J. Antiferromagnetism and the gap of a Mott insulator: Results from analytic continuation of the self-energy. *Phys. Rev. B* **80**, 045101 (2009). URL `http://link.aps.org/doi/10.1103/PhysRevB.80.045101`.

[34] Kaindl, G. *et al.* $4f$ occupation and hybridization from M-shell excitations in rare-earth materials. *Journal of Magnetism and Magnetic Materials* **47-48**, 181 – 189 (1985). URL `http://www.sciencedirect.com/science/article/pii/0304885385903907`.

[35] Allen, J. W., Oh, S. J., Maple, M. B. & Torikachvili, M. S. Large Fermi-level resonance in the electron-addition spectrum of $CeRu_2$ and $CeIr_2$. *Phys. Rev. B* **28**, 5347–5349 (1983). URL `https://link.aps.org/doi/10.1103/PhysRevB.28.5347`.

[36] Smythe, D., Brenan, J., Bennett, N., Regier, T. & Henderson, G. Quantitative determination of cerium oxidation states in alkali-aluminosilicate glasses using $M_{4,5}$-edge XANES. *Journal of Non-Crystalline Solids* **378**, 258 – 264 (2013). URL `http://www.sciencedirect.com/science/article/pii/S0022309313003955`.

[37] Amorese, A. *et al.* 4f excitations in Ce Kondo lattices studied by resonant inelastic x-ray scattering. *Physical Review B* **93**, 1–6 (2016).

[38] Cox, D. L., Bickers, N. E. & Wilkins, J. W. Dynamic magnetic susceptibilities of valence-fluctuation Ce compounds. *Journal of Applied Physics* **57**, 3166–3168 (1985). URL `http://aip.scitation.org/doi/10.1063/1.335137`.

[39] Bickers, N. E., Cox, D. L. & Wilkins, J. W. Self-consistent large-N expansion for normal-state properties of dilute magnetic alloys. *Phys. Rev. B* **36**, 2036 (1987).
